# Supplementary material for: Genome size, genetic diversity, and phenotypic variability imply the effect of genetic variation instead of ploidy on trait plasticity in the cross-pollinated tree species of mulberry
Source: PLoS One. 2023 Aug 11;18(8):e0289766. doi: 10.1371/journal.pone.0289766 (PMC10420377; doi:10.1371/journal.pone.0289766)
Supplement: S1 Fig — (PPTX) [file pone.0289766.s001.PPTX]

## Slide 1
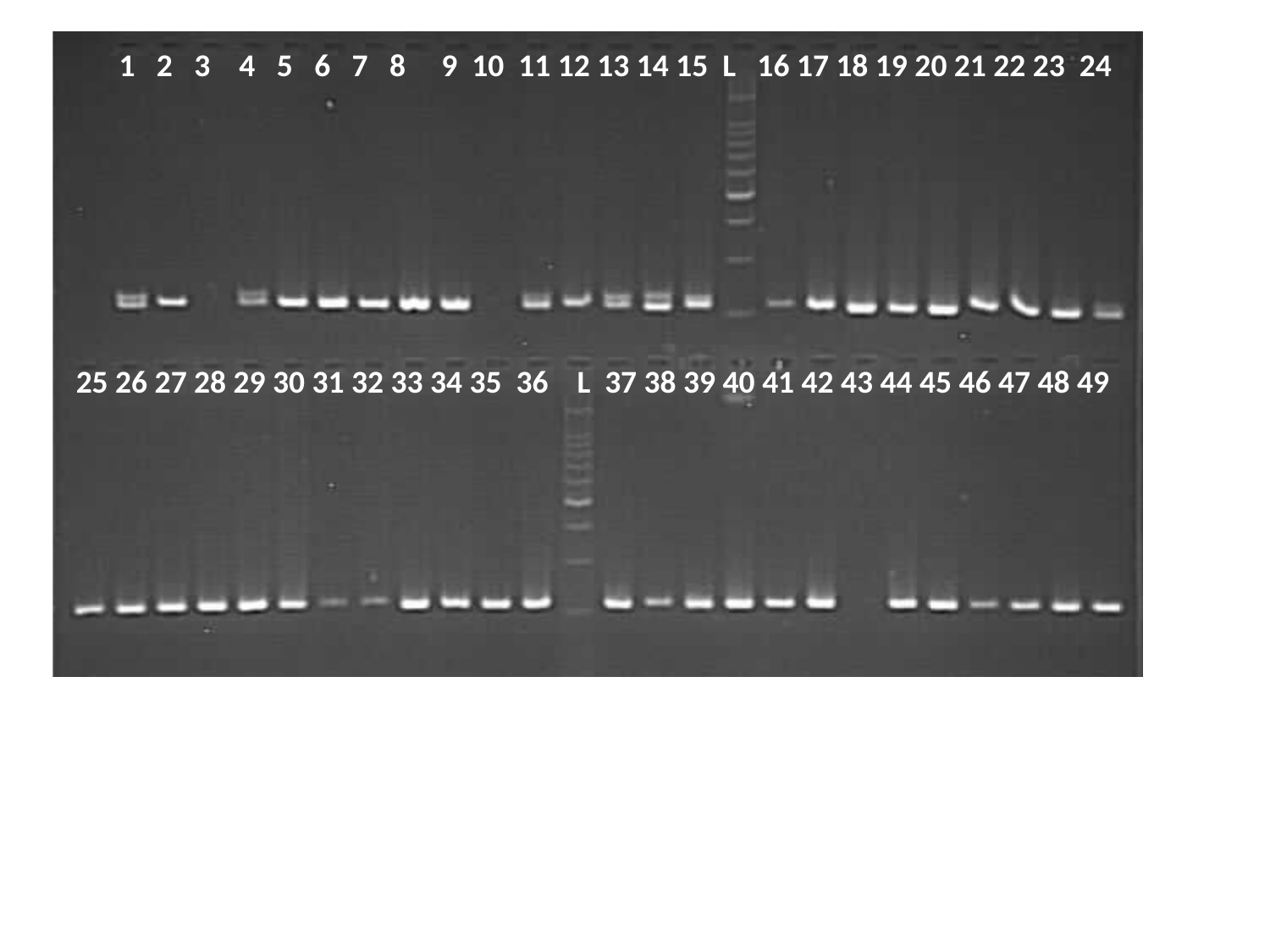

1 2 3 4 5 6 7 8 9 10 11 12 13 14 15 L 16 17 18 19 20 21 22 23 24
25 26 27 28 29 30 31 32 33 34 35 36 L 37 38 39 40 41 42 43 44 45 46 47 48 49

## Slide 2
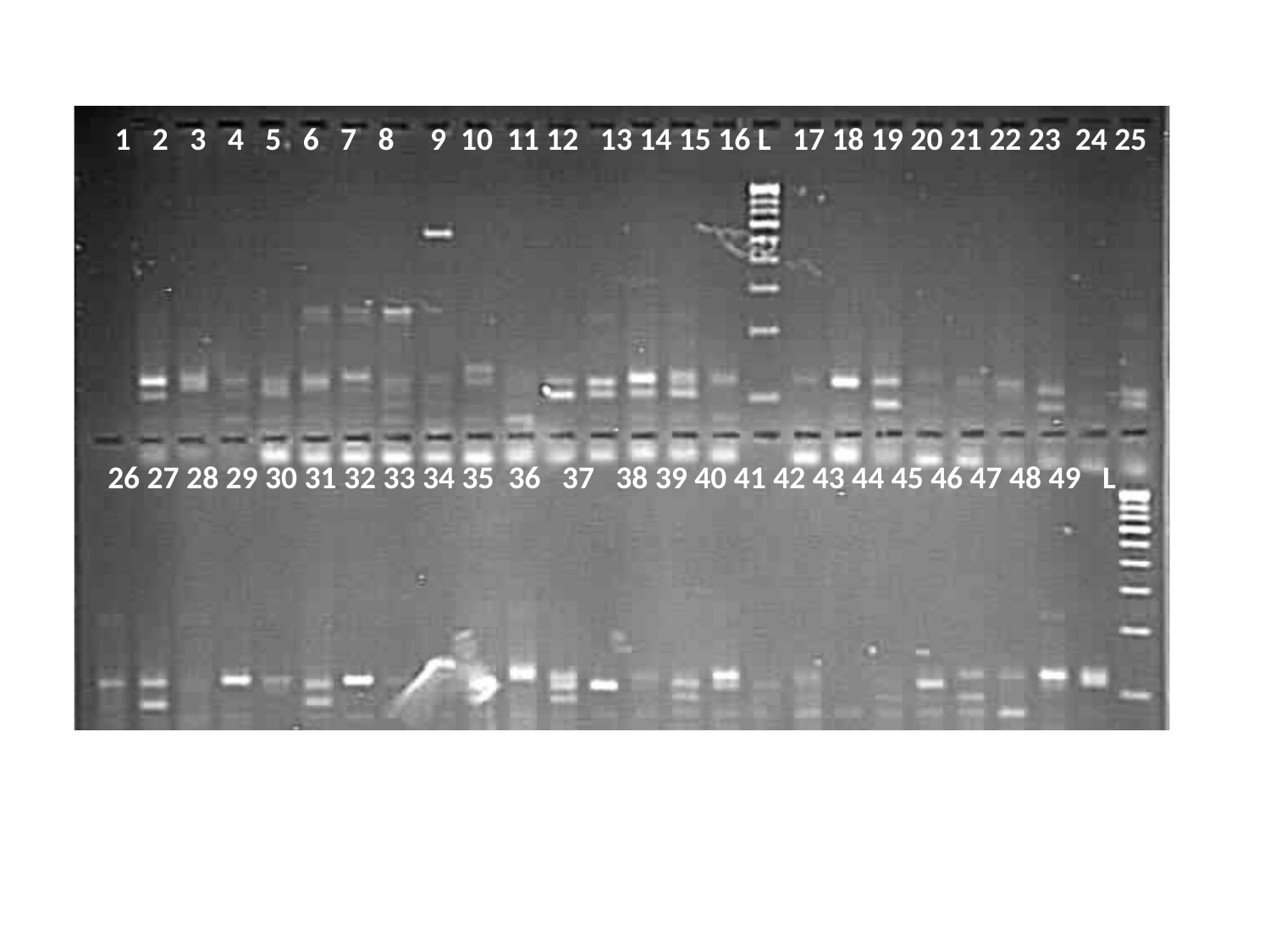

1 2 3 4 5 6 7 8 9 10 11 12 13 14 15 16 L 17 18 19 20 21 22 23 24 25
26 27 28 29 30 31 32 33 34 35 36 37 38 39 40 41 42 43 44 45 46 47 48 49 L

## Slide 3
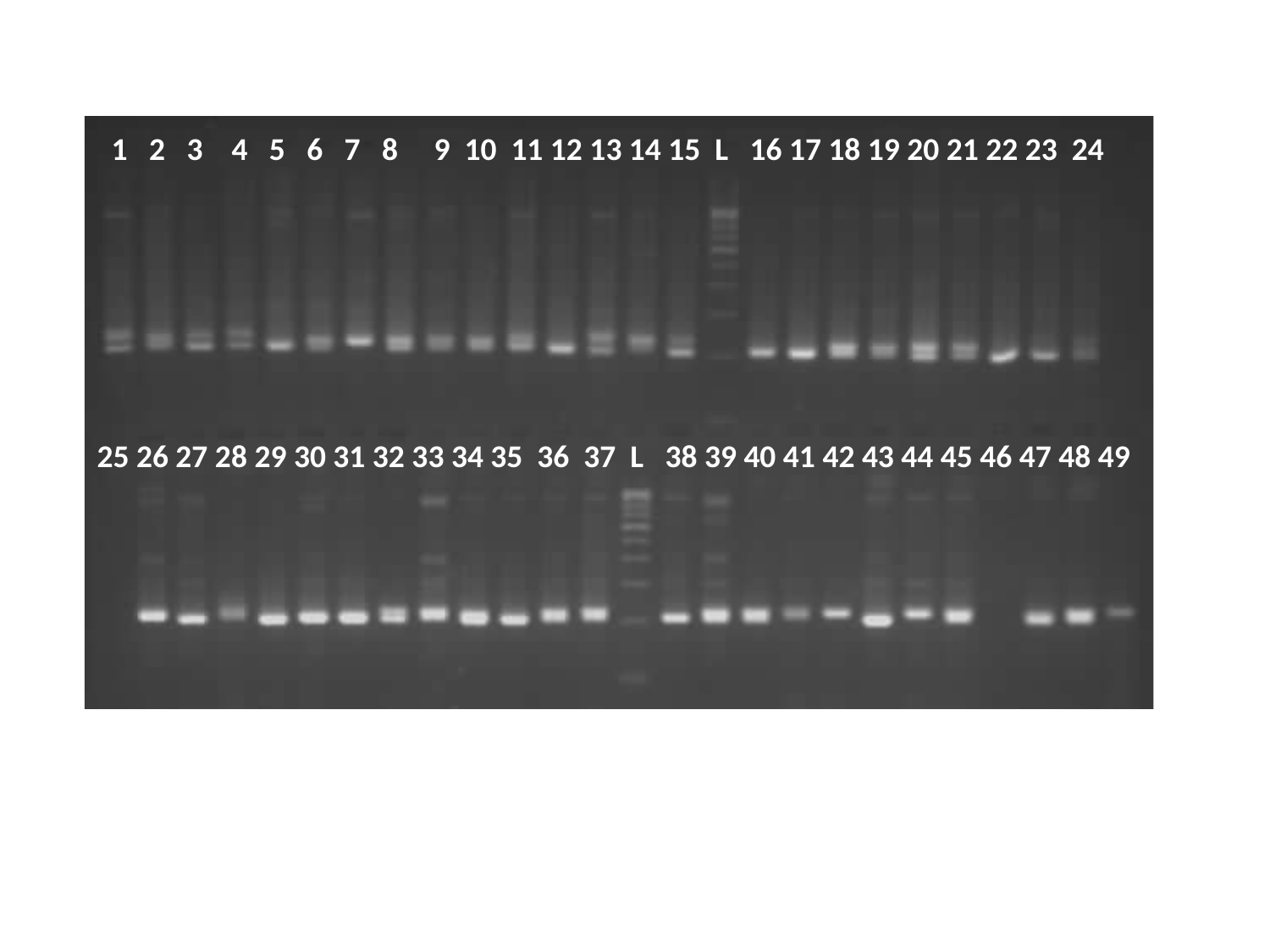

1 2 3 4 5 6 7 8 9 10 11 12 13 14 15 L 16 17 18 19 20 21 22 23 24
25 26 27 28 29 30 31 32 33 34 35 36 37 L 38 39 40 41 42 43 44 45 46 47 48 49

## Slide 4
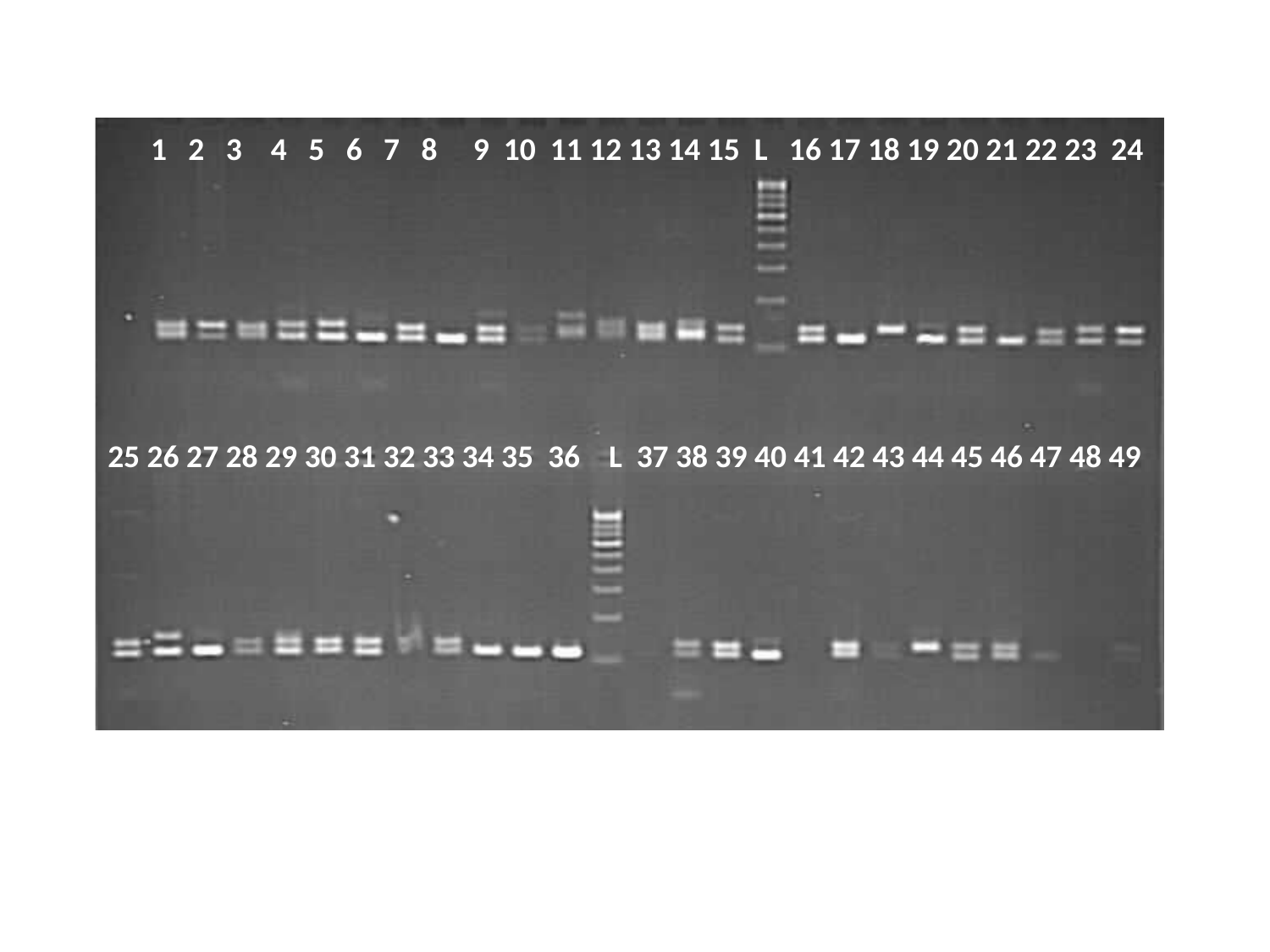

1 2 3 4 5 6 7 8 9 10 11 12 13 14 15 L 16 17 18 19 20 21 22 23 24
25 26 27 28 29 30 31 32 33 34 35 36 L 37 38 39 40 41 42 43 44 45 46 47 48 49

## Slide 5
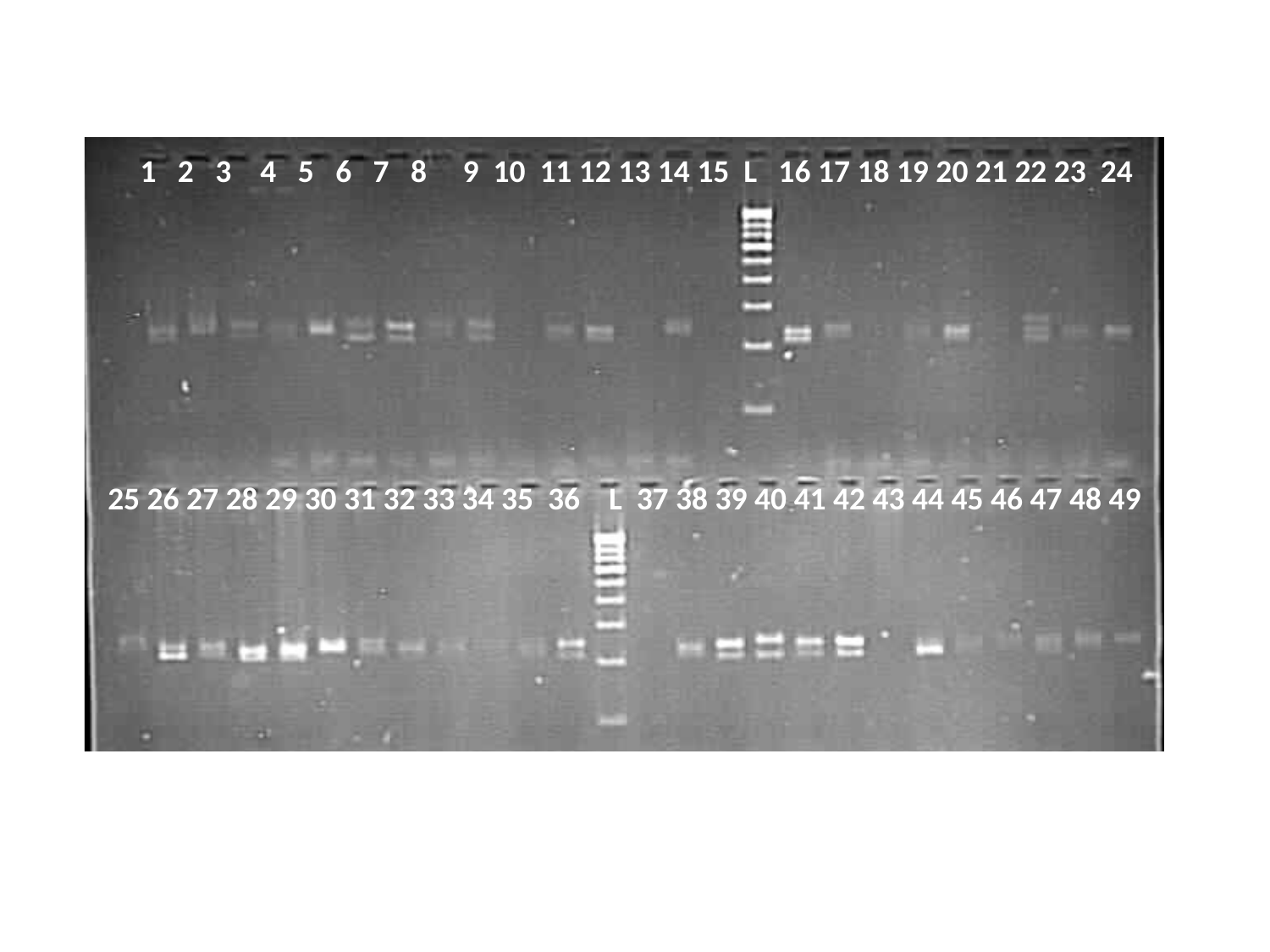

1 2 3 4 5 6 7 8 9 10 11 12 13 14 15 L 16 17 18 19 20 21 22 23 24
25 26 27 28 29 30 31 32 33 34 35 36 L 37 38 39 40 41 42 43 44 45 46 47 48 49

## Slide 6
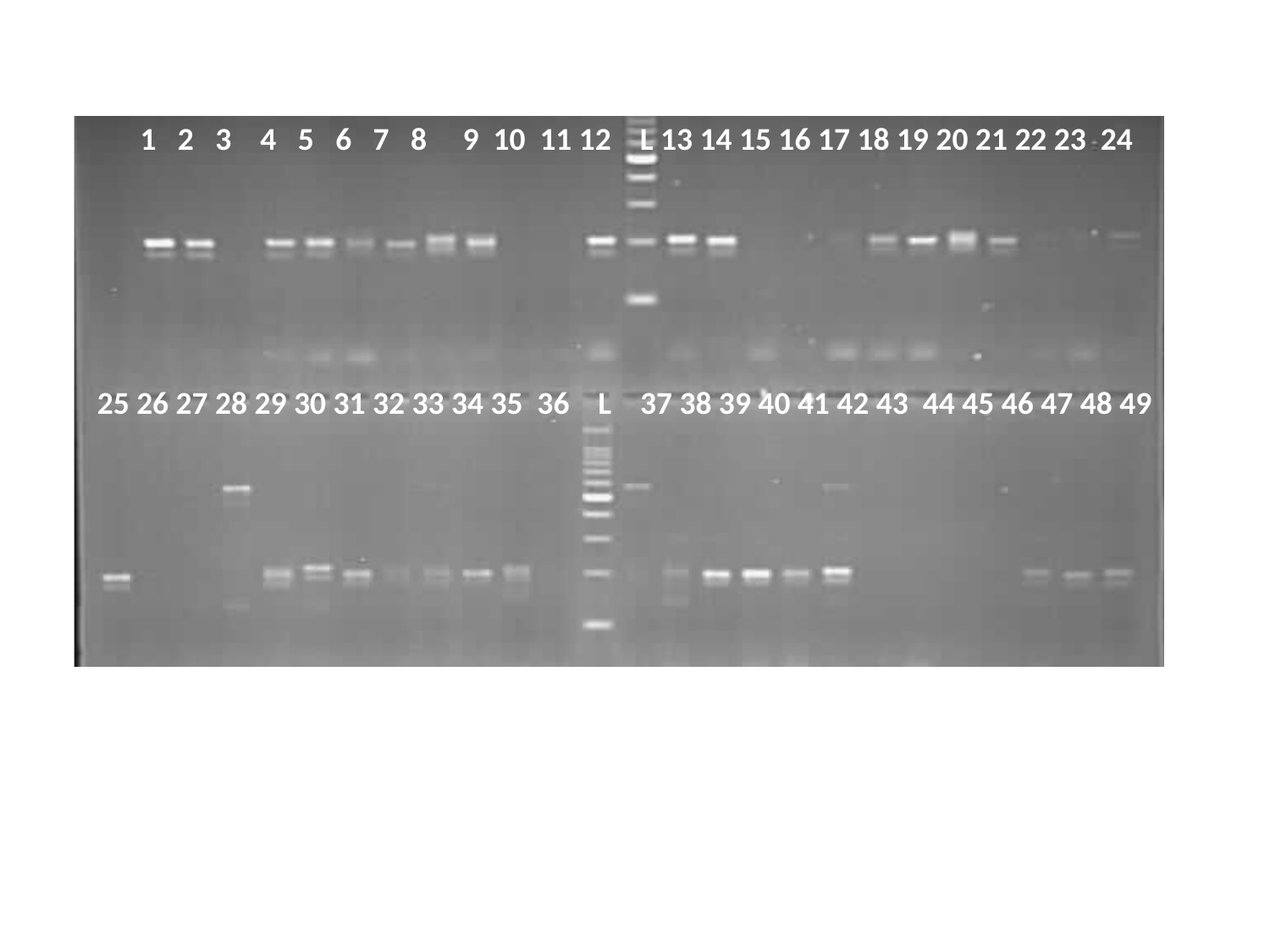

1 2 3 4 5 6 7 8 9 10 11 12 L 13 14 15 16 17 18 19 20 21 22 23 24
25 26 27 28 29 30 31 32 33 34 35 36 L 37 38 39 40 41 42 43 44 45 46 47 48 49

## Slide 7
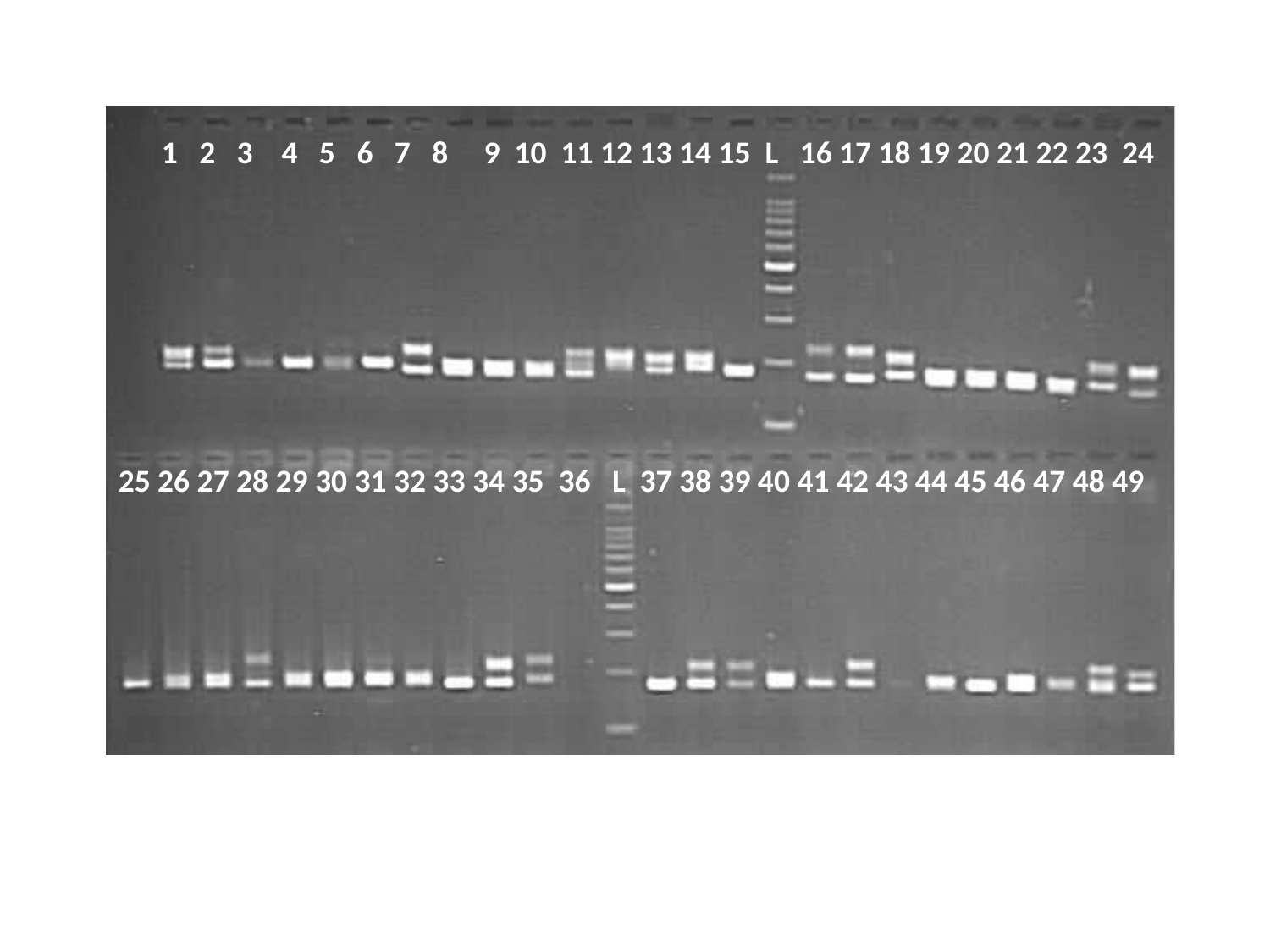

1 2 3 4 5 6 7 8 9 10 11 12 13 14 15 L 16 17 18 19 20 21 22 23 24
25 26 27 28 29 30 31 32 33 34 35 36 L 37 38 39 40 41 42 43 44 45 46 47 48 49

## Slide 8
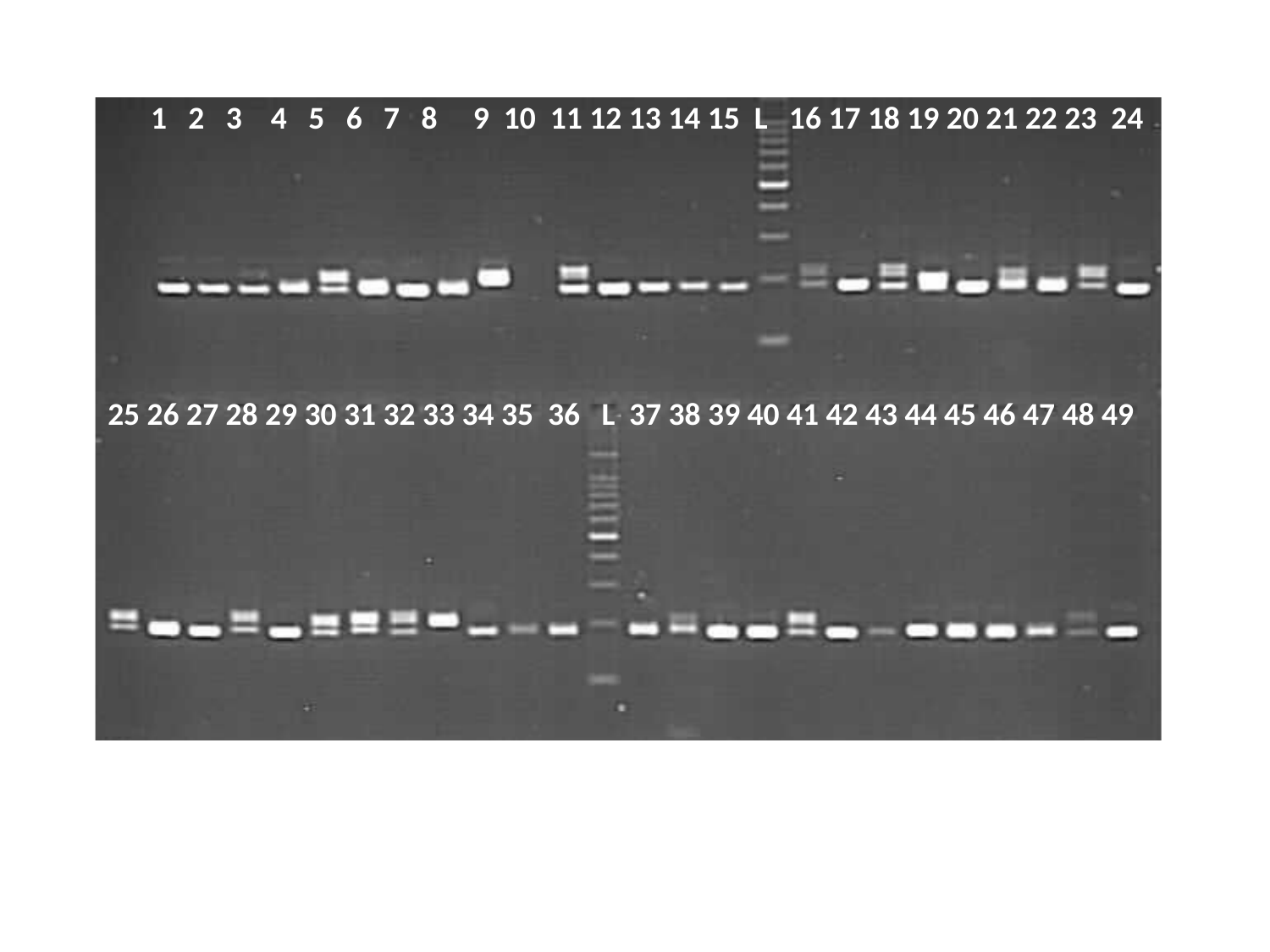

1 2 3 4 5 6 7 8 9 10 11 12 13 14 15 L 16 17 18 19 20 21 22 23 24
25 26 27 28 29 30 31 32 33 34 35 36 L 37 38 39 40 41 42 43 44 45 46 47 48 49

## Slide 9
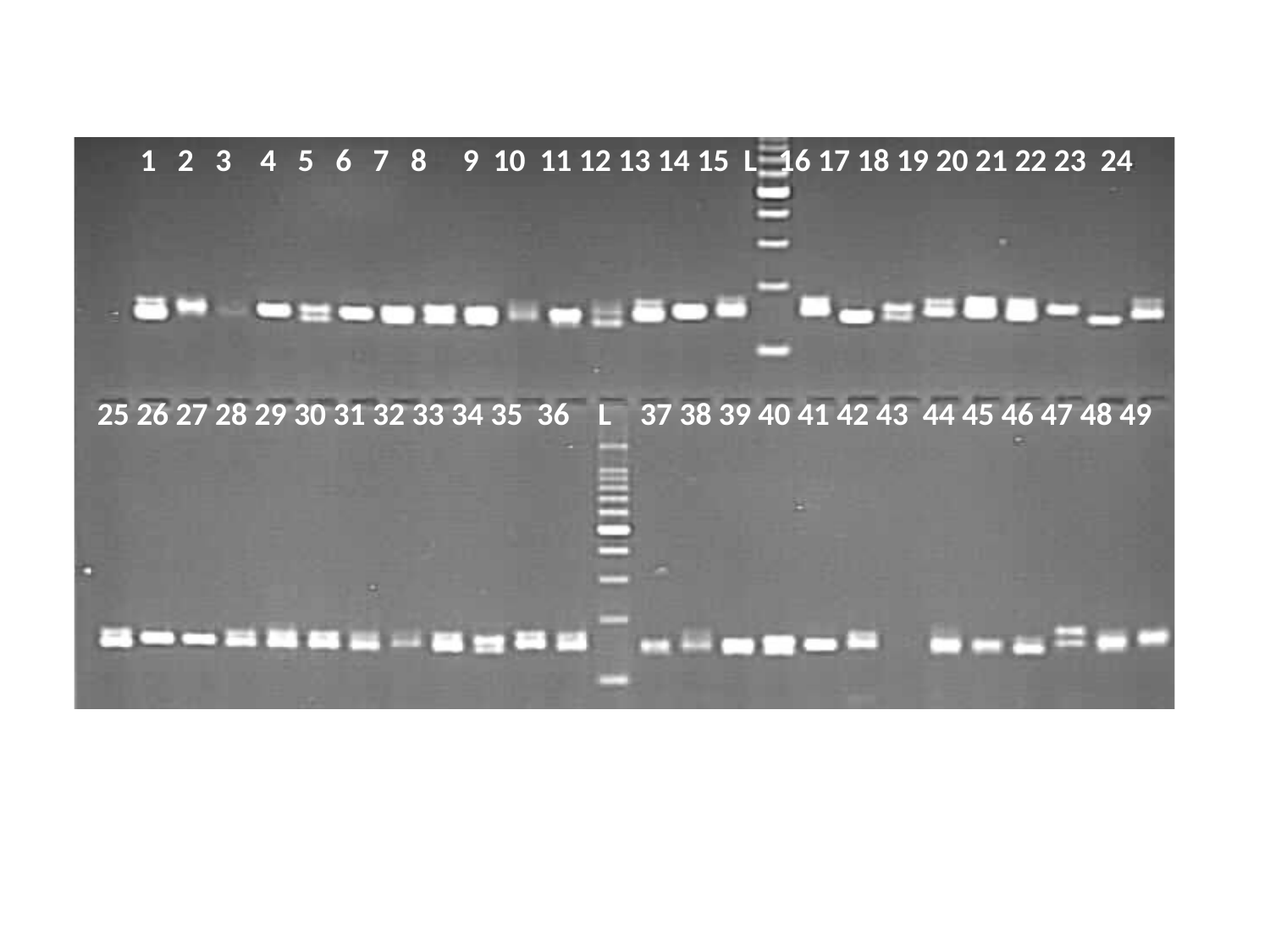

1 2 3 4 5 6 7 8 9 10 11 12 13 14 15 L 16 17 18 19 20 21 22 23 24
25 26 27 28 29 30 31 32 33 34 35 36 L 37 38 39 40 41 42 43 44 45 46 47 48 49

## Slide 10
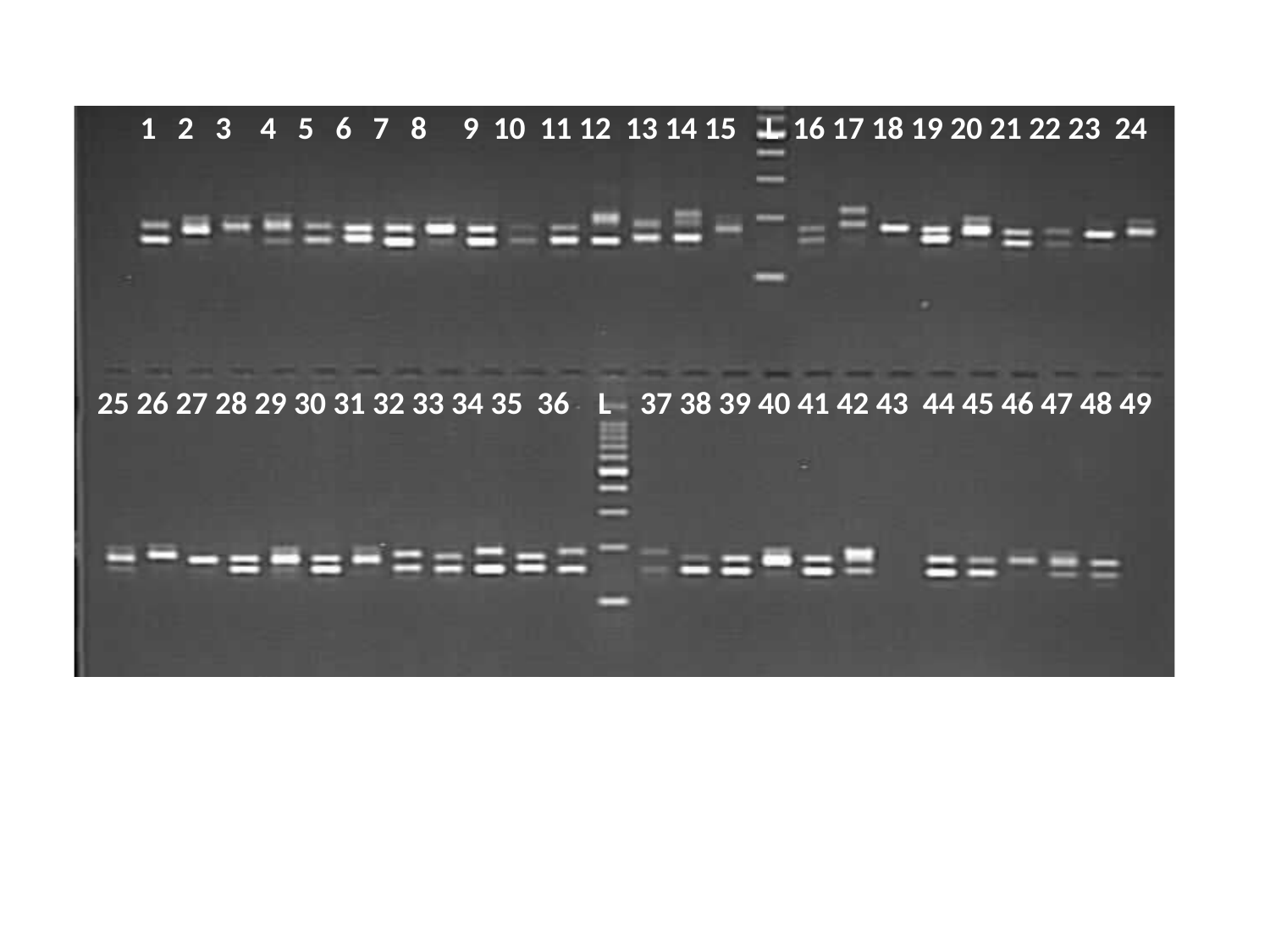

1 2 3 4 5 6 7 8 9 10 11 12 13 14 15 L 16 17 18 19 20 21 22 23 24
25 26 27 28 29 30 31 32 33 34 35 36 L 37 38 39 40 41 42 43 44 45 46 47 48 49

## Slide 11
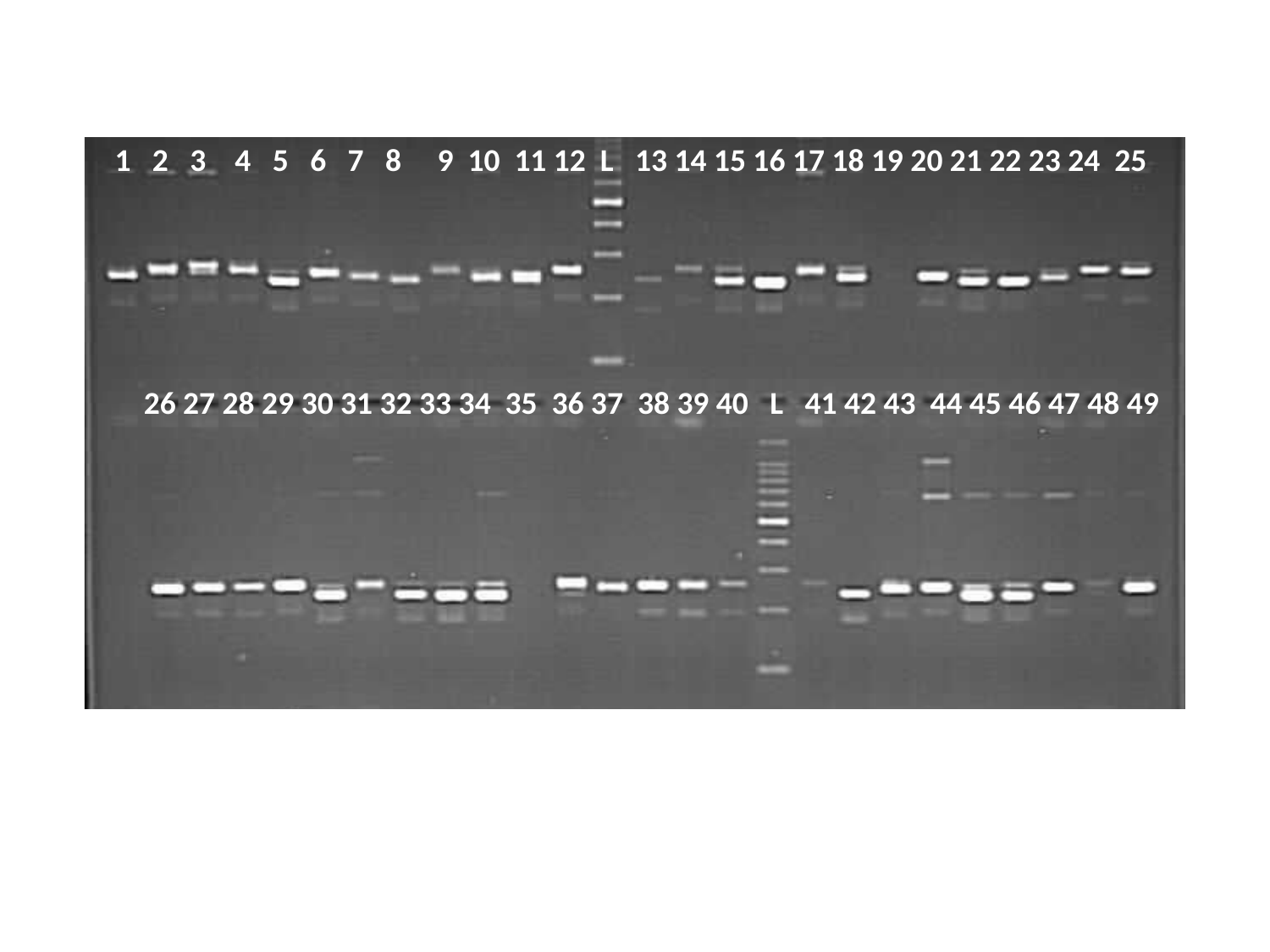

1 2 3 4 5 6 7 8 9 10 11 12 L 13 14 15 16 17 18 19 20 21 22 23 24 25
 26 27 28 29 30 31 32 33 34 35 36 37 38 39 40 L 41 42 43 44 45 46 47 48 49

## Slide 12
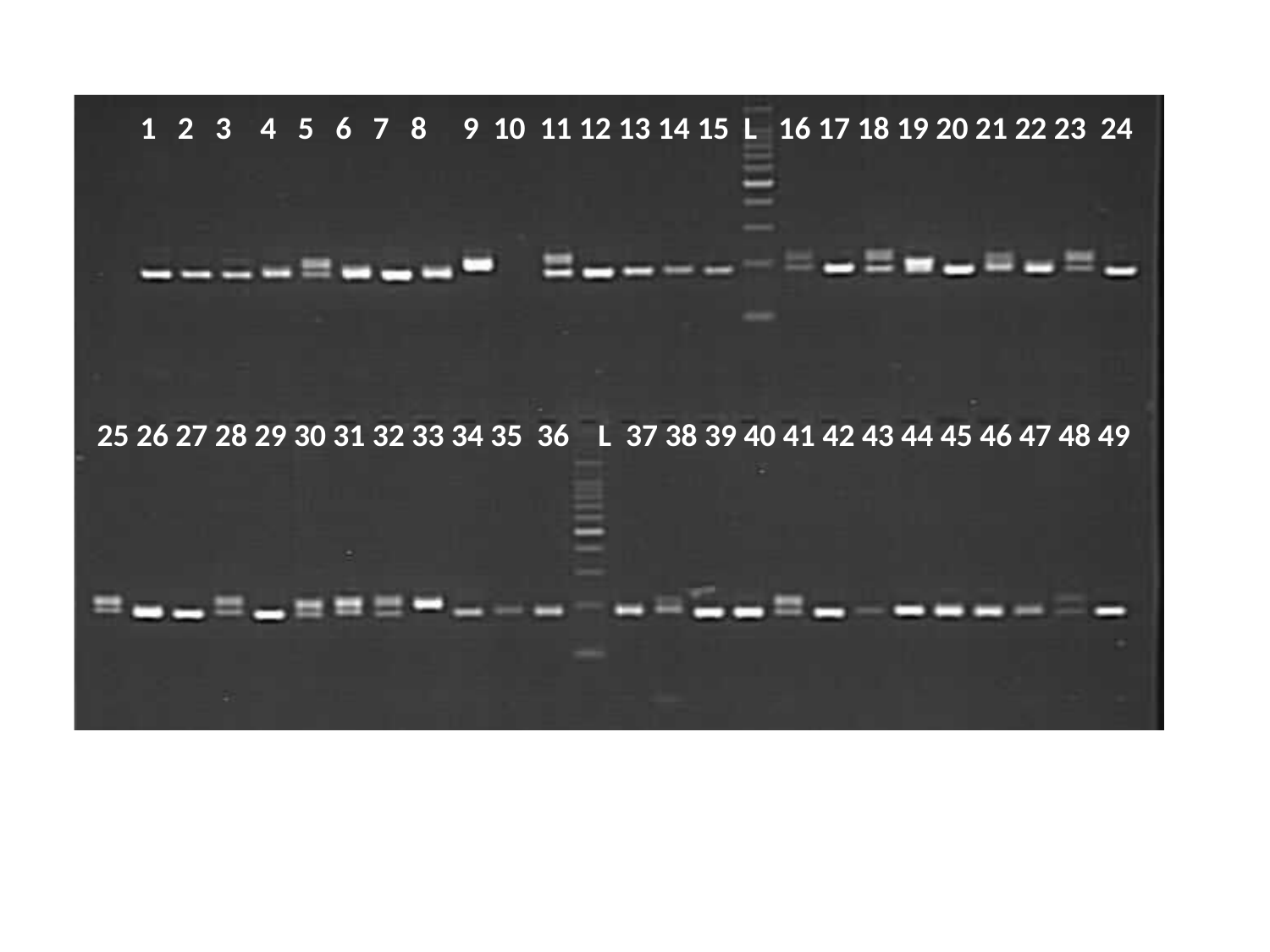

1 2 3 4 5 6 7 8 9 10 11 12 13 14 15 L 16 17 18 19 20 21 22 23 24
25 26 27 28 29 30 31 32 33 34 35 36 L 37 38 39 40 41 42 43 44 45 46 47 48 49

## Slide 13
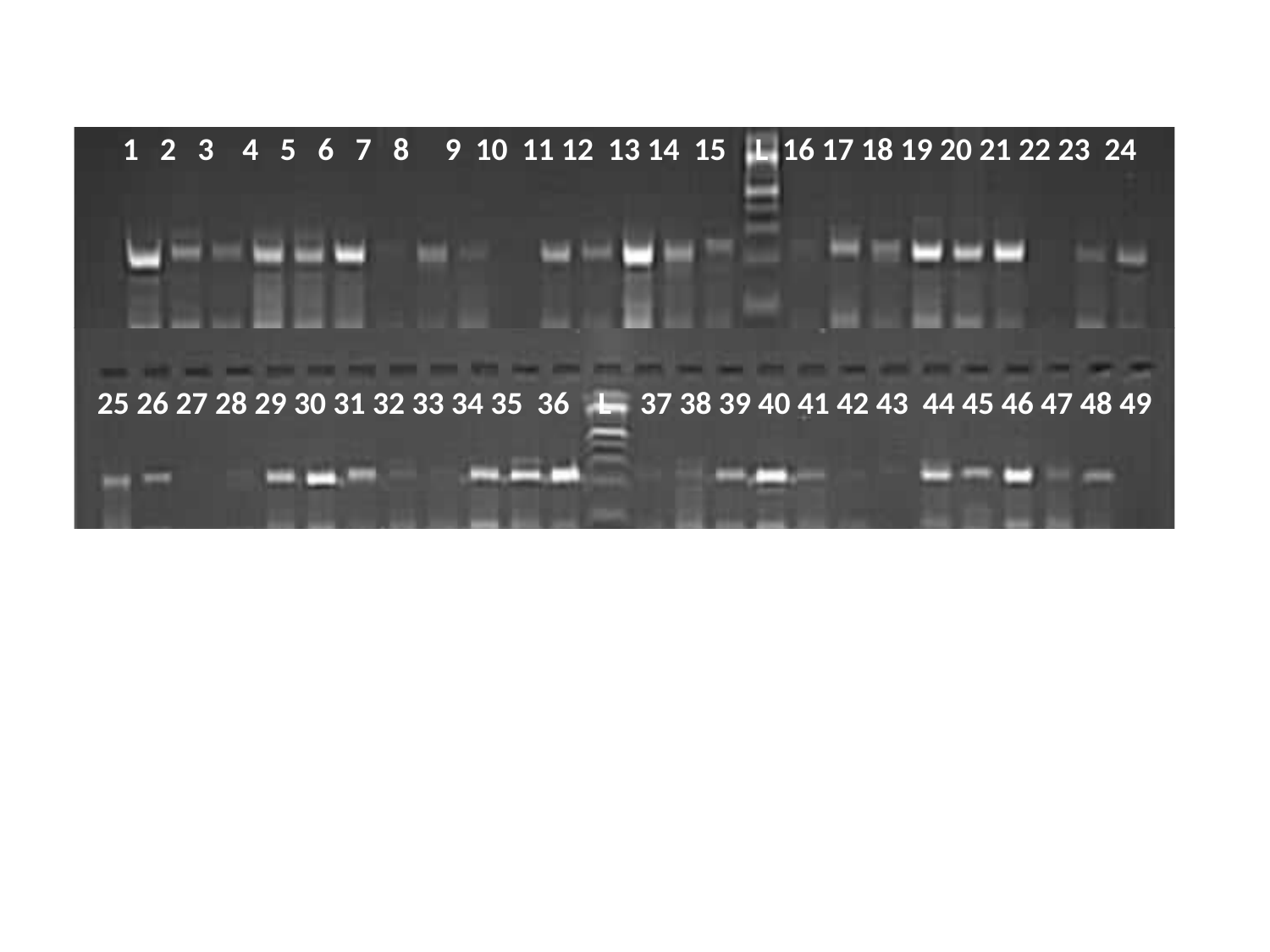

1 2 3 4 5 6 7 8 9 10 11 12 13 14 15 L 16 17 18 19 20 21 22 23 24
25 26 27 28 29 30 31 32 33 34 35 36 L 37 38 39 40 41 42 43 44 45 46 47 48 49

## Slide 14
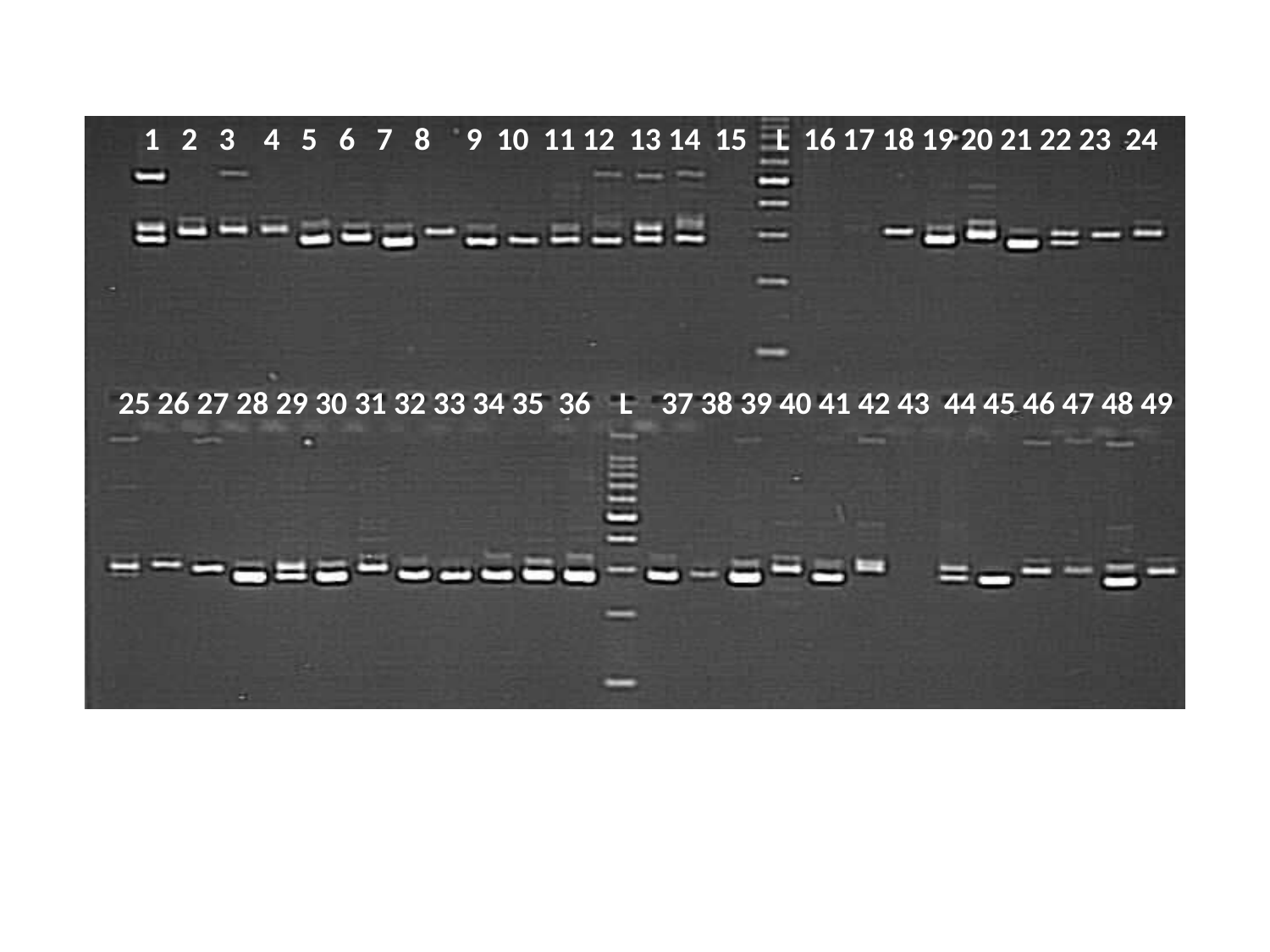

1 2 3 4 5 6 7 8 9 10 11 12 13 14 15 L 16 17 18 19 20 21 22 23 24
25 26 27 28 29 30 31 32 33 34 35 36 L 37 38 39 40 41 42 43 44 45 46 47 48 49

## Slide 15
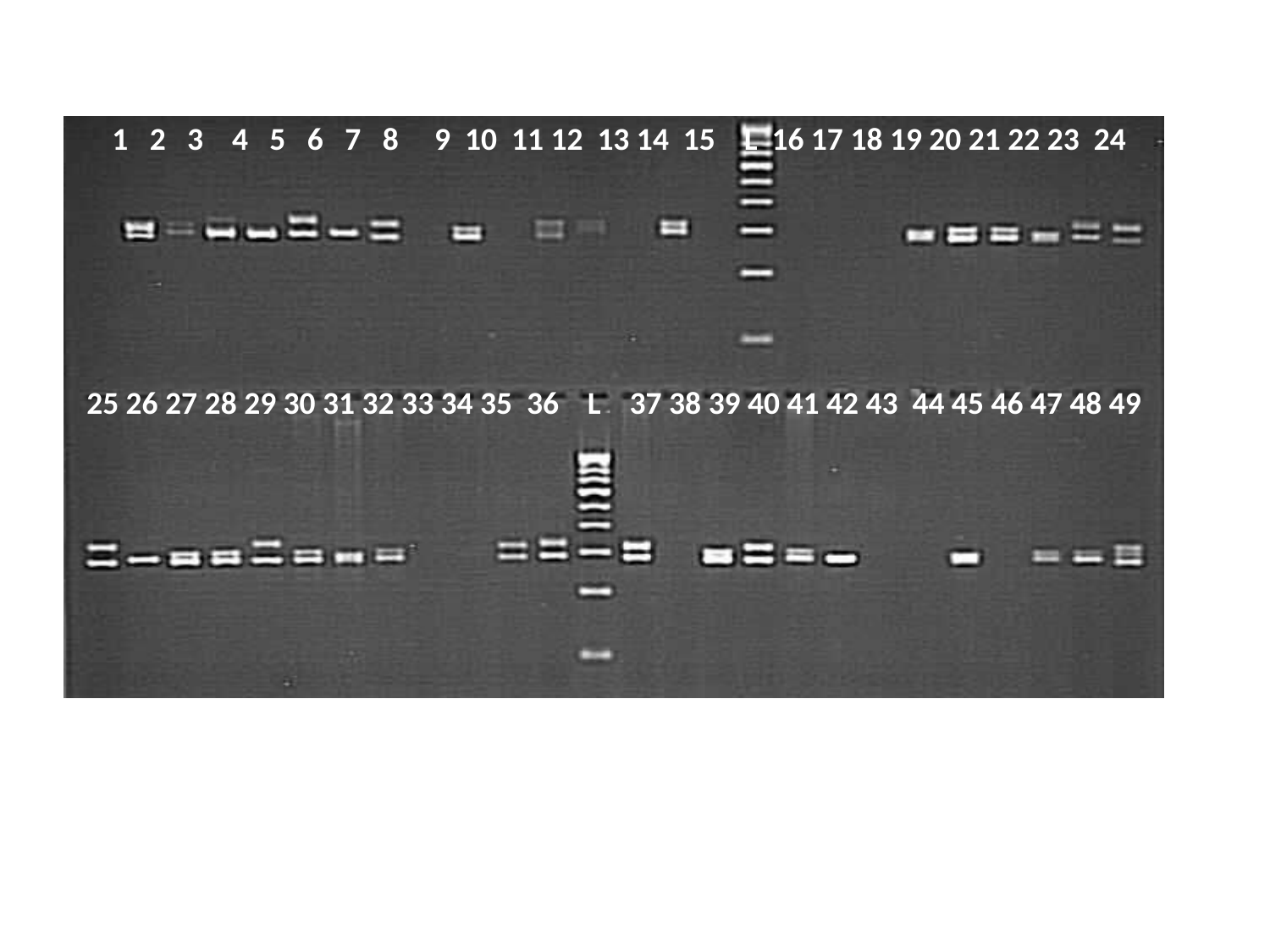

1 2 3 4 5 6 7 8 9 10 11 12 13 14 15 L 16 17 18 19 20 21 22 23 24
25 26 27 28 29 30 31 32 33 34 35 36 L 37 38 39 40 41 42 43 44 45 46 47 48 49

## Slide 16
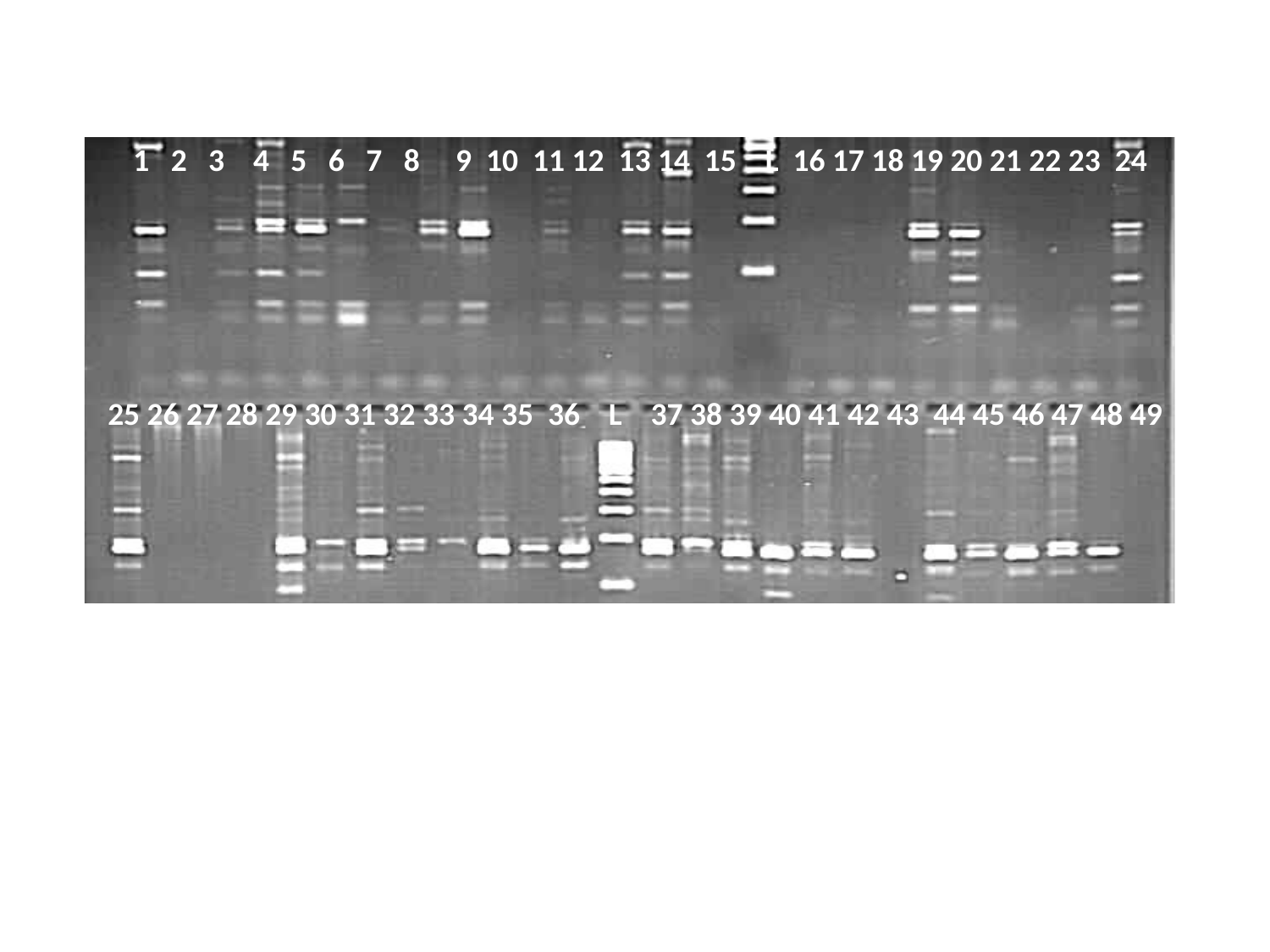

1 2 3 4 5 6 7 8 9 10 11 12 13 14 15 L 16 17 18 19 20 21 22 23 24
25 26 27 28 29 30 31 32 33 34 35 36 L 37 38 39 40 41 42 43 44 45 46 47 48 49

## Slide 17
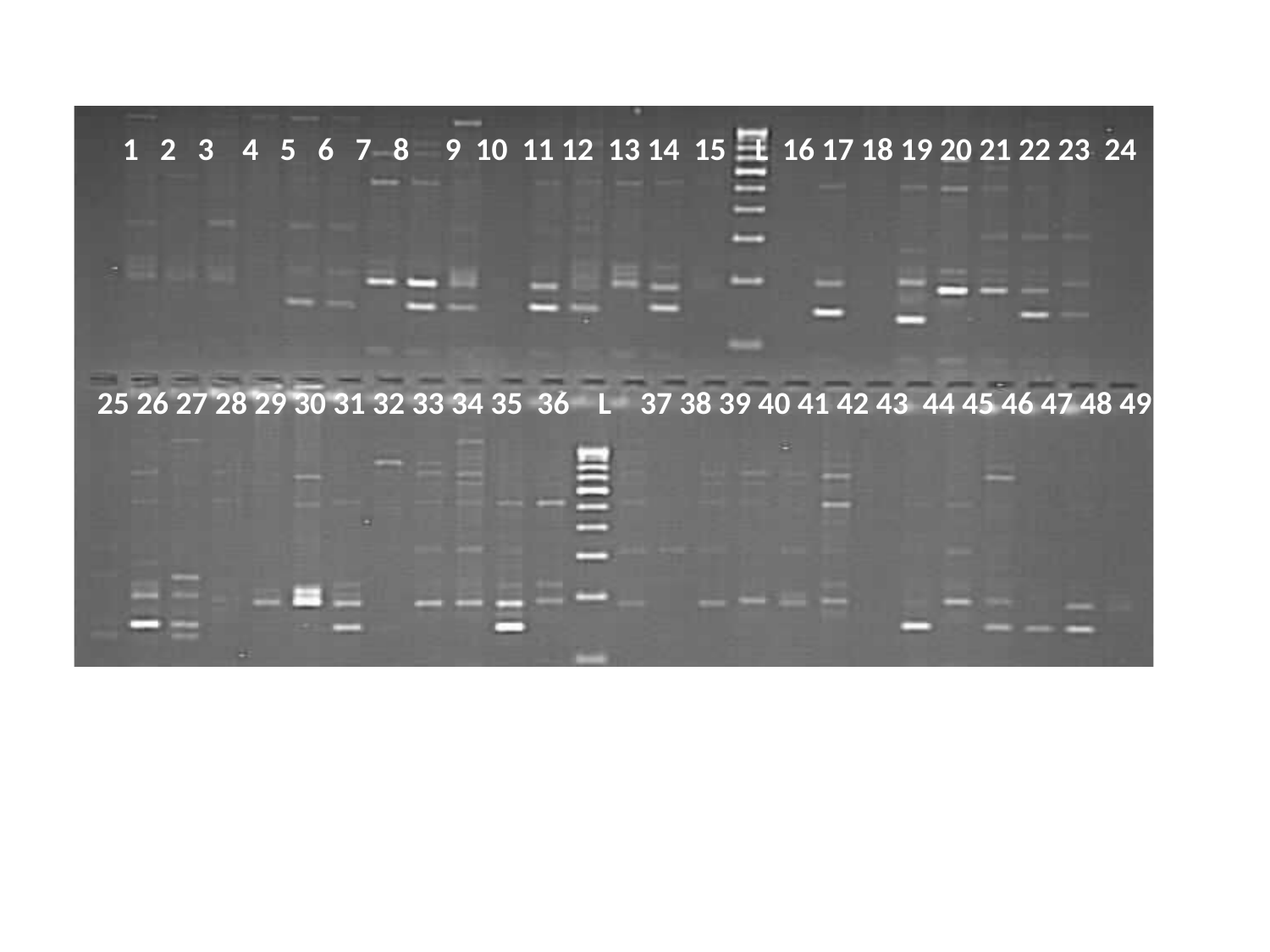

1 2 3 4 5 6 7 8 9 10 11 12 13 14 15 L 16 17 18 19 20 21 22 23 24
25 26 27 28 29 30 31 32 33 34 35 36 L 37 38 39 40 41 42 43 44 45 46 47 48 49

## Slide 18
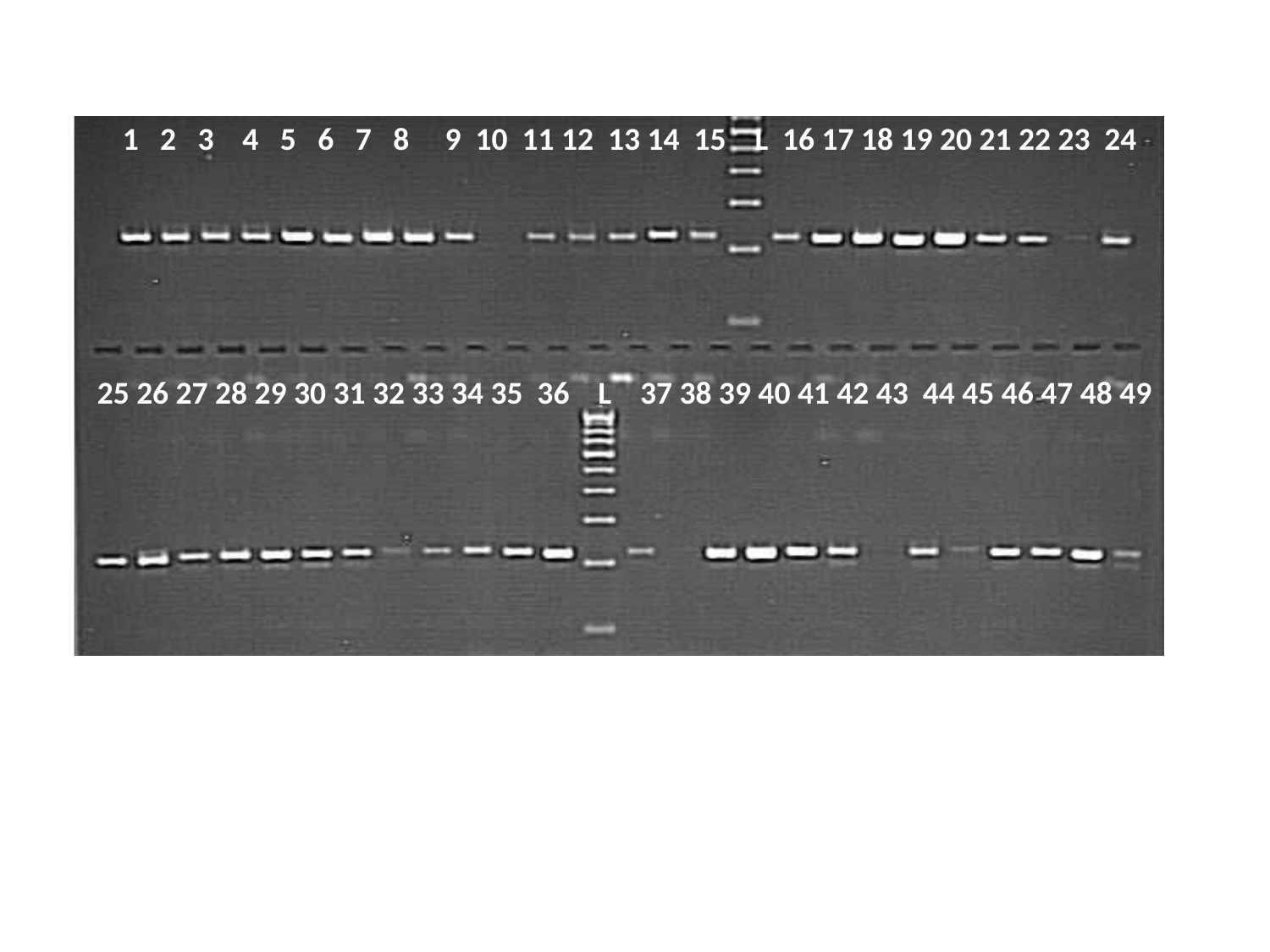

1 2 3 4 5 6 7 8 9 10 11 12 13 14 15 L 16 17 18 19 20 21 22 23 24
25 26 27 28 29 30 31 32 33 34 35 36 L 37 38 39 40 41 42 43 44 45 46 47 48 49

## Slide 19
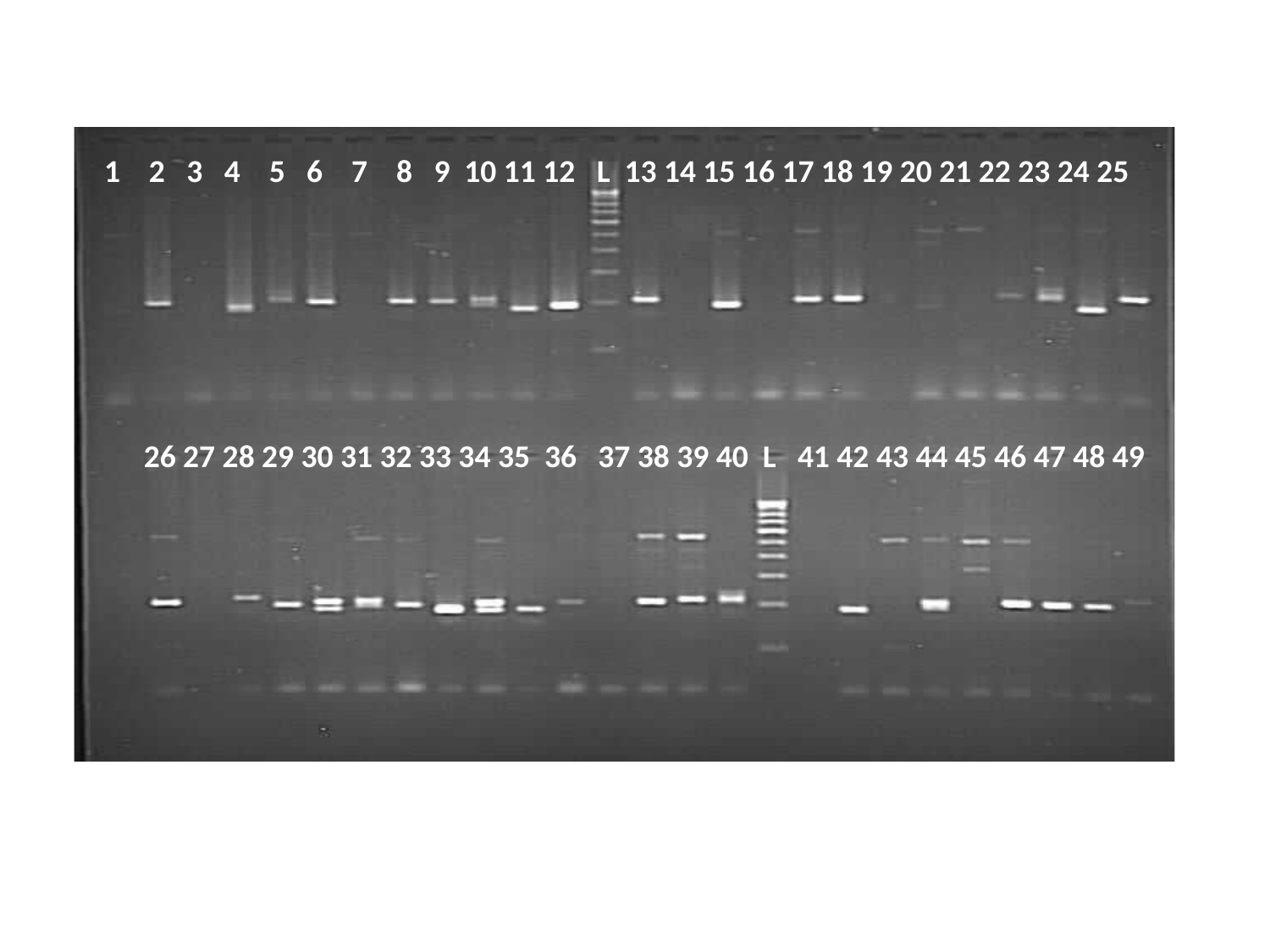

1 2 3 4 5 6 7 8 9 10 11 12 L 13 14 15 16 17 18 19 20 21 22 23 24 25
 26 27 28 29 30 31 32 33 34 35 36 37 38 39 40 L 41 42 43 44 45 46 47 48 49

## Slide 20
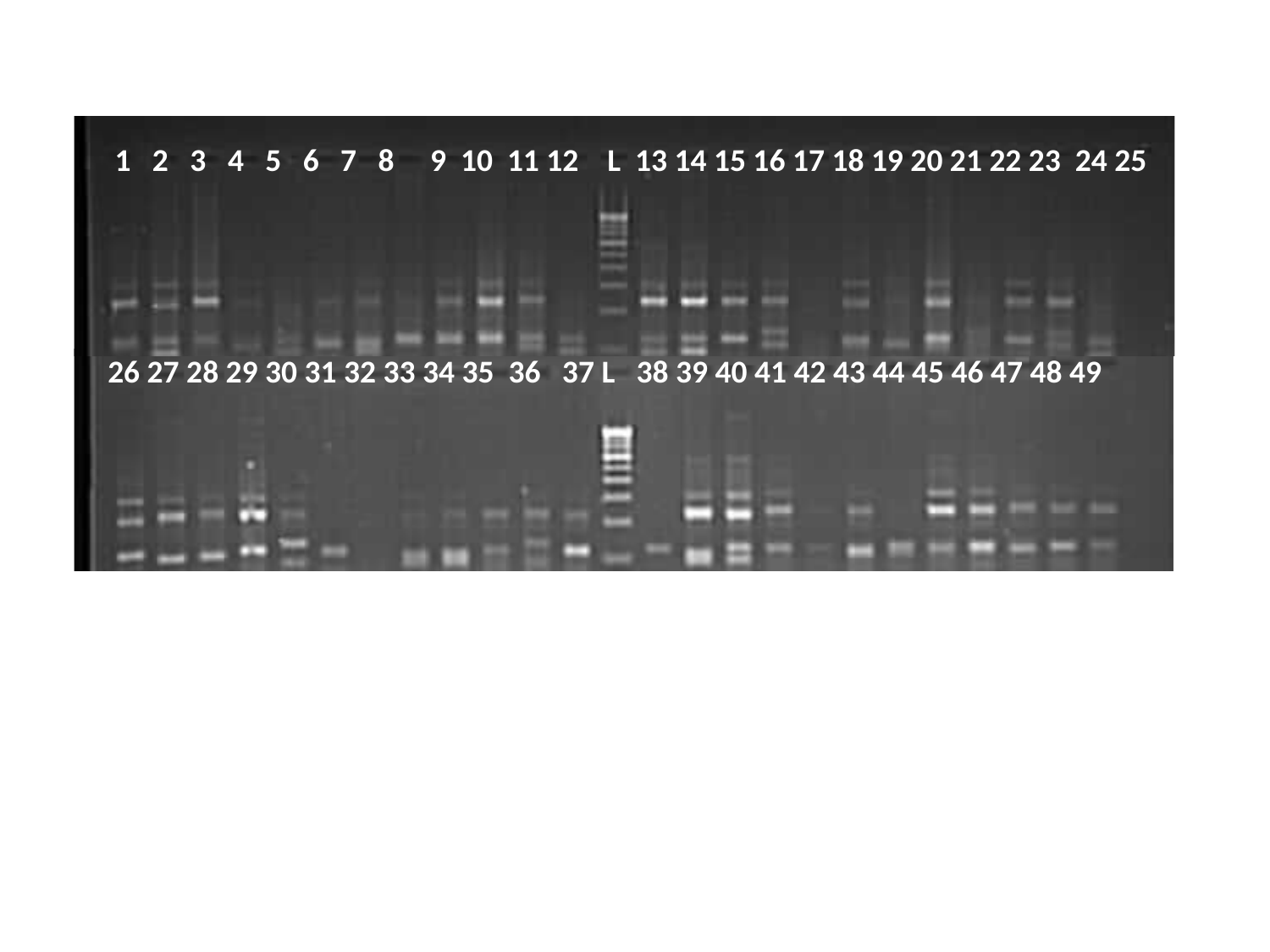

1 2 3 4 5 6 7 8 9 10 11 12 L 13 14 15 16 17 18 19 20 21 22 23 24 25
26 27 28 29 30 31 32 33 34 35 36 37 L 38 39 40 41 42 43 44 45 46 47 48 49
